# RfeA from *Streptococcus suis* serotype 2 triggers NLRP3/Caspase-1-dependent pyroptosis leading to blood–brain barrier disruption

**DOI:** 10.1186/s13567-025-01620-x

**Published:** 2025-09-25

**Authors:** Shuai Gao, Wentao Wu, Xingxing Xiao, Jun Li, Sheng Lei, Luying Wang, Xu Han, Yongliang Lou

**Affiliations:** 1 Wenzhou Key Laboratory of Sanitary Microbiology, Key Laboratory of Laboratory Medicine, Ministry of Education, School of Laboratory Medicine and Life Sciences, Wenzhou Medical University, Wenzhou, 325035 China; 2 Institute of Viral Diseases, Hebei Provincial Center for Disease Control and Prevention, Shijiazhuang, 050021 China; 3 Department of Basic Medical Sciences, Changzhi Medical College, Changzhi, 046000 China

**Keywords:** *Streptococcus suis* serotype 2, RfeA, pyroptosis, NLRP3/Caspase-1, blood–brain barrier

## Abstract

*Streptococcus suis* serotype 2 (SS2) is a prominent pathogen that impacts swine and presents a zoonotic threat to humans; it is a cause of bacterial meningitis, a severe condition linked to neurological impairment and elevated mortality rates. For SS2 to access the central nervous system, it must traverse the blood–brain barrier (BBB); however, the precise mechanisms underlying this process remain incompletely elucidated. In this study, we demonstrate that the RTX family exoprotein A (RfeA), which is secreted by SS2, can be internalized by human brain microvascular endothelial cells (hBMECs) via a caveolae/lipid raft-dependent pathway. RfeA subsequently induces pyroptosis through the NLRP3/Caspase-1 pathway, a process attributed to the increase in mitochondrial reactive oxygen species (mtROS). The interaction between the N-terminus of RfeA and voltage-dependent anion channel 1 (VDAC1) leads to mtROS production, which can be suppressed by a VDAC1 oligomerization inhibitor. RfeA-induced pyroptosis results in disruption of the BBB in both the hBMEC monolayer model and the mouse infection model, thereby promoting bacterial infection of the brain. These findings elucidate a novel mechanism by which SS2 induces pyroptosis to breach the BBB, suggesting a potential target for the prevention and treatment of SS2 infection.

**Supplementary Information:**

The online version contains supplementary material available at 10.1186/s13567-025-01620-x.

## Introduction

*Streptococcus suis* serotype 2 (SS2) is a significant zoonotic pathogen that poses a threat to both swine and human health [1, 2]. The pathogen is known to induce severe diseases, including meningitis, septicemia, and streptococcal toxic shock syndrome (STSS), in both swine and humans [3]. The global distribution of SS2 infections highlights its prevalence in Asia, where it is considered a major cause of bacterial meningitis in adults [4–6]. Notably, two significant outbreaks of SS2 occurred in 1998 and 2005 in Nantong, Jiangsu Province, and Ziyang, Sichuan Province, respectively [7]. Human infections are often linked to occupational exposure, such as pig farming and handling of pork products, as well as the consumption of undercooked pork [8, 9].

To access the central nervous system (CNS), SS2 must cross the blood–brain barrier (BBB), which is composed primarily of brain microvascular endothelial cells (BMECs) [10]. The mechanism by which SS2 crosses the BBB is a complex process involving multiple virulence factors and host‒pathogen interactions. Suilysin, a cholesterol-dependent cytolysin, contributes to the ability of bacteria to cause meningitis by promoting inflammation and apoptosis [11, 12]. SS2 enolase has been identified as a virulence factor that binds to the 40S ribosomal protein SA (RPSA) on the surface of BMECs. This interaction leads to the activation of intracellular signalling pathways, promoting host-apoptosis and increased BBB permeability [13]. The factor H-binding protein (Fhb) of SS2 interacts with globotriaosylceramide, facilitating bacterial traversal across the BBB [14]. Additionally, a serine-rich repeat glycoprotein (SssP1) interacts with vimentin, facilitating bacterial adhesion to and invasion of hBMECs [15]. The role of host immune responses, particularly the production of interleukin-17A (IL-17A), also plays a significant role in the pathogenesis of SS2 meningitis. IL-17A can disrupt the integrity of the blood‒CNS barrier, promoting bacterial invasion and inflammation [16].

Pyroptosis, a form of programmed cell death associated with inflammation, is characterized by the activation of inflammatory caspases, leading to the cleavage of gasdermin D (GSDMD), which forms pores in the cell membrane, resulting in cell swelling, lysis, and the release of cytokines [17]. NOD-like receptor family pyrin domain containing 3 (NLRP3)-mediated pyroptosis is intricately linked to the activation of the NLRP3 inflammasome, a multiprotein complex that plays a crucial role in the innate immune response. The NLRP3 inflammasome is activated in response to a variety of stimuli, including pathogen-associated molecular patterns (PAMPs) and damage-associated molecular patterns (DAMPs) [18, 19]. The mechanism of NLRP3 activation involves three primary signalling pathways: the reactive oxygen species pathway, the lysosomal pathway, and the ion channel pathway [20–23]. Upon lysosomal damage, cathepsin B is released into the cytosol, where it can activate the NLRP3 inflammasome [24]. When activated by extracellular ATP, the purinergic 2X7 receptor (P2X7R) facilitates the assembly of the NLRP3 inflammasome [25]. Mitochondria are a significant source of reactive oxygen species (ROS), which are byproducts of the electron transport chain [26].

Recent studies have highlighted the role of pyroptosis in the pathogenesis of SS2 infections. For example, SS2 can activate the NLRP3 inflammasome, which in turn activates Caspase-1, leading to the cleavage of GSDMD and subsequent pyroptotic cell death [27]. This process is crucial for the inflammatory response during SS2 infection and contributes to the severity of the disease [28]. Virulence factors of SS2, such as suilysin, have been implicated in inducing pyroptosis [29, 30]. A recent study indicated that SS2-induced NLRP3 activation contributes to BBB disruption [31]. Membrane vesicles isolated from SS2 also trigger pyroptosis in endothelial cells through the activation of the NLRP3/Caspase-1/GSDMD signalling pathway [32].

Targeting the pathways involved in pyroptosis, such as the NLRP3 inflammasome or Caspase-1, could improve the clinical outcomes of SS2 infections. RfeA, a member of the RTX (repeats-in-toxin) family, has been identified in SS2. Purified recombinant RfeA has been shown to elicit protective immunity in animal models, suggesting its potential role as a virulence factor or immunogen [33], but its specific functions in SS2 pathogenesis remain unknown. We hypothesize that RfeA plays a role in helping SS2 break through the BBB. Here, we discovered that RfeA secreted by SS2 interacts with VDAC1 to induce pyroptosis, ultimately leading to disruption of the BBB.

## Materials and methods

### Cell culture and transfection

Human brain microvascular endothelial cells (hBMECs) were purchased from ScienCell Research Laboratories. The cells were maintained in Dulbecco’s modified Eagle’s medium (DMEM) supplemented with 10% fetal bovine serum (Gibco) under conditions of 5% CO_2_ at 37 °C.

Transfections of hBMECs were conducted using Lipofectamine 3000 (Thermo Fisher) in OptiMEM (Gibco), which incorporates 2 μg of plasmid DNA in a 1 mL reaction mixture. Following a 12-h incubation period, the medium was replaced with fresh medium supplemented with 10% foetal bovine serum.

### Inhibitor and protein treatment

For experiments involving chemical inhibitors, hBMECs were pretreated separately with chlorpromazine (Selleck, 15 µg/mL, 1 h), wortmannin (MCE, 200 nM, 1 h), calmidazolium (MCE, 10 µM, 1 h), glibenclamide (MCE, 100 µM, 0.5 h), Z-YVAD-FMK (MCE, 5 µM, 0.5 h), CA-074me (MCE, 10 µM, 1 h), AZ10606120 (MCE, 10 µM, 1 h), Mito Tempo (MCE, 250 µM, 1 h), or VBIT-12 (MCE, 10 µM, 6 h).

For functional assessment of RfeA, 2 × 10^5^ hBMECs were treated with 10 µg/mL purified recombinant RfeA variants (wild-type, ΔN-terminus, or N-terminus) for 4 h. For time-dependent response analysis, the cells were exposed to purified proteins at 0, 1, 2, 4, 6, and 8 h post-stimulation. The control cells were treated with solvent (vehicle) alone.

### Bacterial strains

The *S. suis* serotype 2 strain 05ZYH33, which was isolated from a patient with meningitis, was used for *rfea* cloning and to establish a mouse infection model. The strain was cultured in Todd-Hewitt broth (Hopebio) supplemented with 1% yeast extract (Oxoid) at 37 °C.

For the generation of the Δ*rfea* mutant strain, the genomic DNA of strain 05ZYH33 was used as a template. The upstream and downstream arms of the gene were obtained by PCR amplification with the primers Δrefa-A/B and Δrfea-C/D, respectively. Fusion PCR was performed with the primers Δrfea-A/D, and the product was subsequently cloned and inserted into the plasmid pSET4s. The details of the primers used are provided in Additional file 1. The pSET4s-Δrfea strain was electrotransferred into 05ZYH33 competent cells, and the bacteria were subsequently grown on TH agar supplemented with 100 μg/mL spectinomycin at 37 °C. The colonies that subsequently developed on the agar were inoculated and propagated in THB at 28 °C over the course of 10 generations. The knockout strains exhibited susceptibility to spectinomycin, a condition verified through PCR analysis.

### Plasmid construction

Genomic DNA from the *S. suis* serotype 2 05ZYH33 strain was isolated using the Bacteria Genomic DNA Extraction Kit (TaKaRa). The sequences encoding RfeA-WT, the RfeA-N-terminus (1–27 aa) and the RfeA-ΔN-terminus (deletion of 1–27 aa) were subsequently amplified via PCR via Rapid Taq Master Mix (Vazyme Biotech) employing specific primers, as detailed in Additional file 1. The purified fragments were ligated to pEGFP-C1, pET-28a, or pcDNA3.1 via T4 DNA ligase (TaKaRa).

### Protein purification

The constructed pET28a vectors, which were ligated with RfeA-WT, RfeA-ΔN-terminus, or RfeA-N-terminus, were transformed into *Escherichia coli* BL21(DE3) cells, which were subsequently cultured in Luria–Bertani (LB) broth supplemented with 50 μg/mL kanamycin at 37 °C. The expression of the recombinant proteins was induced with 1 mM isopropyl-β-d-thiogalactoside (IPTG) at 16 °C for 12 h. Following induction, the bacterial cells were harvested by centrifugation and subjected to lysis. The lysate was then centrifuged, and Ni-TED Sepharose (Sangon Biotech) was used to purify the proteins from the supernatant.

### Immunofluorescence

A total of 2 × 10^5^ cells were seeded on glass coverslips (WHB Scientific) placed in 12-well plates. Following the addition of 10 μg/mL rRfeA for 2 h or transfection, the cells were fixed with 4% paraformaldehyde for 20 min at 25 ℃. The paraformaldehyde was subsequently removed, and the cells were incubated with QuickBlock blocking and permeabilization buffer (Beyotime). Next, the slides were incubated with rabbit anti-His monoclonal antibodies (CST, 1:200) diluted in immunofluorescent primary antibody dilution buffer (Beyotime) at 4 °C for 12 h with gentle shaking. The cells were finally incubated with YSFluor^™^ 488 goat anti-rabbit lgG (H + L) secondary antibodies (Yeasen, 1:200) in immunofluorescent secondary antibody dilution buffer (Beyotime) for 2 h at 25 °C. Nuclear staining was performed with 10 µg/mL DAPI solution (Beyotime) for 15 min at room temperature. Images were obtained by confocal microscopy (Nikon).

### CCK8 assay

BMECs (2 × 10^5^ h) were treated with 10 µg/mL rRfeA for 0–8 h. Subsequently, 10 μL of CCK-8 solution was added to each well, and the cells were incubated at 37 °C for an additional 2 h. Optical density was then measured at a wavelength of 450 nm.

### LDH assay

Following seeding in a 12-well cell culture plate, 2 × 10^5^ hBMECs were exposed to rRfeA at a concentration of 10 µg/mL for 0–8 h. Subsequently, the cell culture media was collected and analysed in accordance with the protocol provided by the Lactate Dehydrogenase Assay Kit (Nanjing Jiancheng Bioengineering Institute).

### Spectrophotometric haemolytic assay

rRfeA at a final concentration of 10 μg/mL was added to a suspension of 1.5% sheep erythrocytes in PBS buffer containing 20 mM MgCl_2_. The mixture was incubated at 37 °C for 90 min, followed by centrifugation at 3000 × *g* for 5 min. Subsequently, the absorbance of each sample was measured at a wavelength of 550 nm using a microplate reader.

### Western blot

Following a 5-min boiling period in loading buffer, the samples were subjected to SDS‒polyacrylamide gel electrophoresis. The proteins were subsequently transferred to PVDF membranes (Bio-Rad) at a constant current of 300 mA for 90 min. The membranes were then blocked with a 5% (w/v) BSA solution prior to incubation with primary antibodies, which were diluted in specific antibody dilution buffer (Beyotime). The primary antibodies used included rabbit anti-caspase-1 p20 polyclonal antibodies (Abmart, 1:500), rabbit anti-NLRP3 monoclonal antibodies (CST, 1:1000), rabbit anti-mCherry monoclonal antibodies (ABclonal, 1:1000), rabbit anti-HA monoclonal antibodies (CST, 1:1000), rabbit anti-β-Tubulin monoclonal antibodies (Proteintech, 1:1000), and rabbit anti-His monoclonal antibodies (CST, 1:1000). The membranes were further incubated with horseradish peroxidase (HRP)-conjugated goat anti-rabbit IgG antibodies (Beyotime, 1:1000) or HRP-conjugated goat anti-mouse IgG antibodies (Beyotime, 1:1000). The activity of HRP was detected using Enhanced Chemiluminescence Western Blotting Substrate (GE).

### ELISA

To assess the level of IL-1β secreted by hBMECs, the cell culture supernatant was analysed using an ELISA kit (ABclonal) in accordance with the manufacturer’s instructions. Specifically, 100 µL of sample was added to each well of the provided adhesive strip. Following incubation, the wells were emptied, and 100 µL of biotin antibody and 100 µL of 1 × HRP-avidin were sequentially added. After the addition of the TMB substrate, the reaction was subsequently terminated, and the optical density of each well was measured within 5 min using a microplate reader set to 450 nm.

### mtROS measurement

BMECs (2 × 10^5^ h) were seeded in glass-bottom culture dishes and incubated for 12 h. The cells were exposed to 10 µg/mL rRfeA for 2 h or 4 h and then coincubated with 10 µM mitoSOX (Yeasen) in DMEM for 30 min at 37 °C. Confocal microscopy was used to spatially quantify the mean fluorescence intensity (MFI) of mtROS. For flow cytometry analysis, cells were gated on live singlets using FSC and SSC, and the fluorescence intensity was analysed at the single-cell level.

### Coimmunoprecipitation (Co-IP) and LC‒MS/MS analysis

hBMECs (2 × 10^7^) were transfected with hemagglutinin (HA)-tagged RfeA either individually or in combination with mCherry-VDAC1. Twenty-four hours post-transfection, the cells were lysed with 2 mL of immunoprecipitation lysis buffer (Beyotime) containing protease inhibitors (Roche). The lysed cell samples were then centrifuged at 12 000 × *g* at 4 °C for 25 min, and the supernatant was collected. Subsequently, 20 µL of anti-HA antibody-coated agarose (Lablead) was added, and the mixture was incubated with rotation at 4 °C for 2 h. Following centrifugation at 2655 × *g* 3 times, 60 µL of 2 × SDS loading buffer was added to the samples, which were then boiled at 95 °C for 10 min to elute the proteins. For identification of proteins that interact with RfeA, samples were subsequently sent to the Beijing Genomics Institute (BGI) for further processing and identification. Briefly, the samples were subjected to SDS‒PAGE, dehydrated with acetonitrile, and digested with trypsin. Peptides were separated via an UltiMate 3000 UHPLC (Thermo Fisher) and analysed via a Q Exactive HF tandem mass spectrometer (Thermo Fisher).

### In vitro BBB model construction and assay

Transwell chambers (1.12 cm^2^, 3 µm pore size) were coated with collagen type I (Sigma) at a concentration of 10 µg/cm^2^. The collagen coating was allowed to dry overnight. Five hundred microliters of hBMECs were seeded at a density of 1 × 10^5^ cells/mL onto the apical side of the Transwell chambers. The culture mixture was maintained in DMEM to equilibrate the internal and external liquid levels of the culture plates, and the cells were cultured for a period of 7–10 days to establish complete monolayers. To investigate the impact of RfeA on barrier integrity, hBMEC monolayers were incubated with 10 µg/mL rRfeA for 0–6 h. Subsequently, the Transwell inserts were transferred to a new plate containing Hanks’ balanced salt solution (HBSS) in the lower chamber, and 50 µL of 0.4% Evans blue solution in PBS was added to the upper chamber. The Transwell inserts were incubated at 37 °C with 5% CO_2_ for 40 min. Permeability was evaluated through colorimetric quantification by measuring the optical density at 600 nm (OD_600_) in the lower chamber.

### Animal experiments

Five-week-old C57BL/6 J mice were obtained from Zhejiang Vital River Laboratory Animal Technology Co. Ltd. and maintained under specific pathogen-free (SPF) conditions. Twenty-four female mice were randomly assigned to 4 groups (*n* = 6 per group). Each mouse received an intraperitoneal injection of 5 × 10^8^ CFU of either the WT strain or the Δ*rfea* strain, which was subsequently suspended in an equal volume of sterile phosphate-buffered saline (PBS). Two days post-infection, brain samples were collected under sterile conditions, and the tissues were homogenized in PBS, followed by serial dilution and plating on TSA. The plates were incubated at 37 °C for 48 h, after which colony-forming units (CFU) were enumerated. For the evaluation of BBB permeability, 100 µL of 2% Evans blue (EB) was administered intravenously 48 h post-infection. After 2 h, the brains were excised, photographed, and subjected to desiccation at 56 °C in aluminum foil for 2 days. Formamide was used to extract Evans blue from the tissue, and the quantity of EB was quantified by measuring the absorbance at an optical density of 620 nm (OD_620_).

### Quantification and statistics

All the quantitative experiments were conducted with a minimum of three biologically independent replicates. Confocal microscopy image quantification was carried out on at least 50 images from each group. Statistically significant differences were determined using one-way, two-way analysis or t tests with Prism 9 software.

## Results

### RfeA is endocytosed by hBMEC in a caveolae/lipid raft-dependent manner

**Figure 1:**
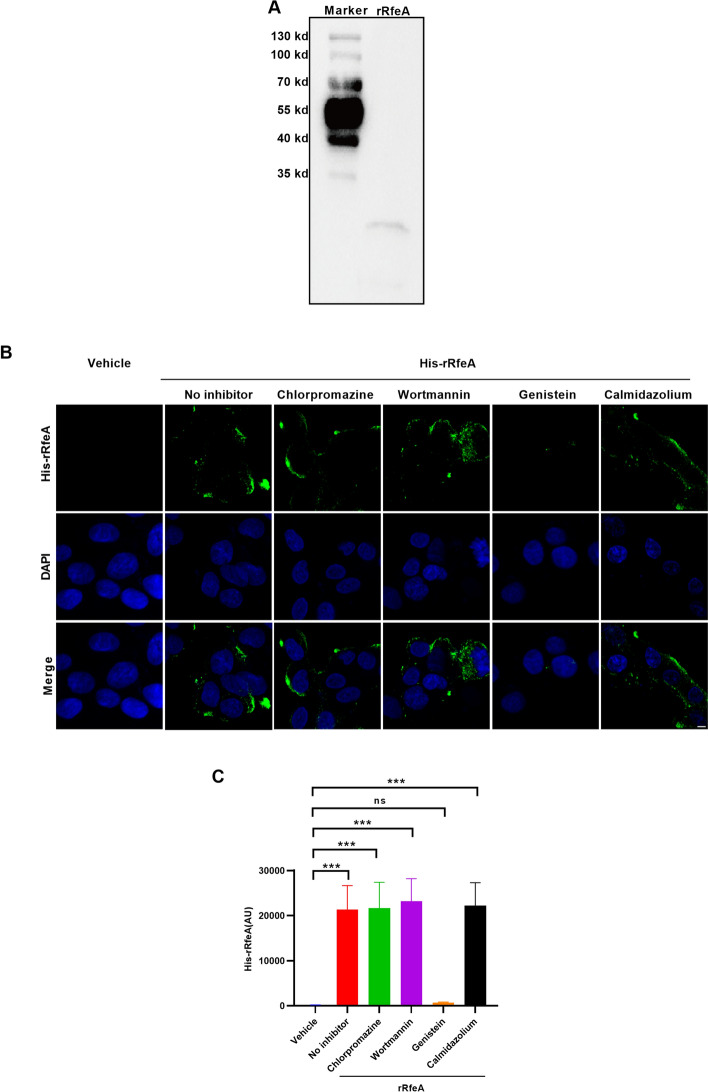
**RfeA internalization into hBMECs requires caveolae/lipid raft-dependent endocytosis. A** Western blot validation of purified His-rRfeA. The left lane displays the marker, whereas the right lane shows the rRfeA band at approximately 22 kDa. **B** Representative confocal microscopy images showing rRfeA internalization in hBMECs treated with different inhibitors: chlorpromazine, wortmannin, genistein or calmidazolium. Control cells were treated with vehicle (PBS). The cell nucleus was stained with DAPI. Scale bar: 5 μm. **C** Quantitative analysis of rRfeA internalization efficiency by means of fluorescence intensity measurements. X-axis: different groups; Y-axis: MFI (arbitrary units). The data are presented as the means ± s.d.; *n* = 51 images. Significant differences in comparison with the vehicle control were identified by one-way ANOVA followed by Dunnett’s multiple comparisons test. ***P < 0.001.

The majority of bacterial toxins require cellular entry to exert their toxic effects. To determine whether RfeA can be internalized by hBMECs, we expressed recombinant RfeA (rRfeA) with a His tag in *E. coli* BL21 (DE3) followed by purification and identification via western blot analysis with anti-His antibodies. As depicted in Figure 1A, a distinct and prominent band was observed at the specified position (approximately 22 kDa). rRfeA was incubated with hBMECs for 2 h, after which the cells were fixed and probed with anti-His antibodies. Confocal microscopy revealed a strong intracellular immunofluorescence signal of His-rRfeA, whereas hBMECs not treated with rRfeA presented no positive signal, indicating that RfeA is indeed capable of entering hBMECs (Figures 1B, C). To further elucidate the pathway involved in rRfeA internalization, hBMECs were treated with chlorpromazine, genistein, or wortmannin, which are known inhibitors of clathrin-dependent endocytosis, caveolae/lipid raft-dependent endocytosis, and macropinocytosis, respectively [34]. Previous studies have reported that the internalization of the well-characterized RTX family exoprotein CyaA is dependent on calmodulin [35]; therefore, calmidazolium, a calmodulin inhibitor, was also used to pretreat hBMECs. Our results revealed that genistein treatment significantly reduced the amount of rRfeA internalized into hBMECs, but chlorpromazine, wortmannin and calmidazolium had no effect on the endocytosis of rRfeA, which illustrated that rRfeA entered hBMECs via caveolae/lipid raft-dependent endocytosis (Figures 1B, C).

### RfeA induces pyroptosis through the NLRP3/Caspase-1 signalling pathway

**Figure 2:**
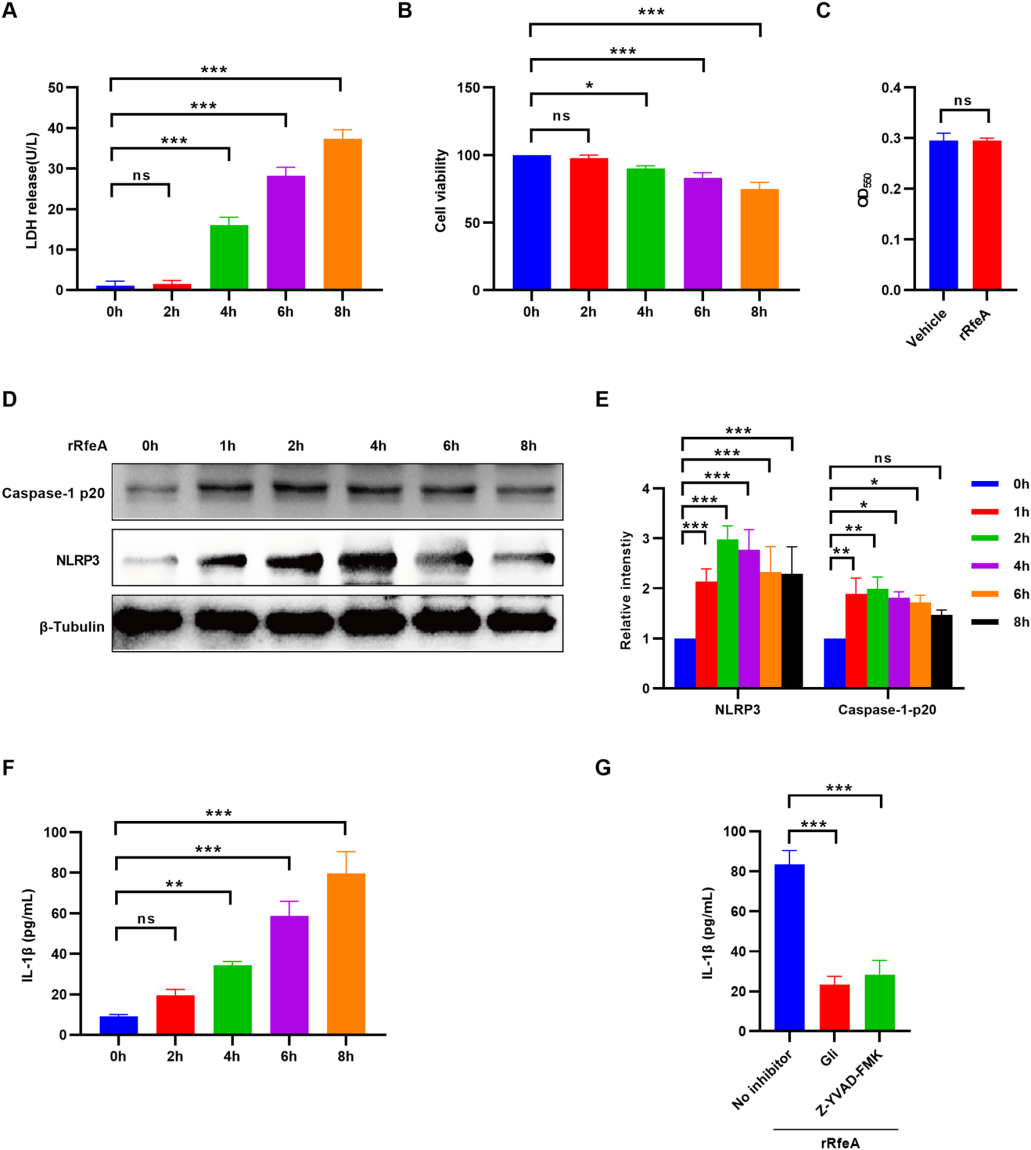
**RfeA induces pyroptosis in hBMECs via the NLRP3/Caspase-1 pathway**. **A** LDH levels released by hBMECs treated with rRfeA for 0–8 h. The data are presented as the means ± s.d.; *n* = 3. Significant differences compared with 0 h were identified via one-way ANOVA followed by Dunnett’s multiple comparisons test. ***P < 0.001. **B** Cell viability was determined by a CCK8 assay at 0–8 h post-rRfeA treatment. The data are presented as the means ± s.d.; *n* = 3. Significant differences compared with 0 h were identified using a one-way ANOVA followed by Dunnett’s multiple comparisons test. *P < 0.05; ***P < 0.001. **C** Assessment of the hemolytic activity of rRfeA by spectrophotometry. The increase in the OD_550_ is correlated with sheep erythrocyte lysis. The data are presented as the means ± s.d.; *n* = 3. **D** Representative western blots of NLRP3, Caspase-1 p20, and β-Tubulin (loading control) in rRfeA-treated hBMECs. Samples were collected at 0–8 h post-rRfeA treatment. **E** Quantitative analysis of the intensities of the NLRP3 and Caspase-1 p20 protein bands in Panel D. 0 h was used as a reference (1.0). The data are presented as the means ± s.d.; *n* = 3. Significant differences compared with 0 h were identified by two-way ANOVA followed by Dunnett’s multiple comparisons test. *P < 0.05; **P < 0.01; ***P < 0.001. **F** Measurement of IL-1β secreted by hBMECs in culture supernatants following treatment with rRfeA for 0–8 h by ELISA. The data are presented as the means ± s.d.; *n* = 3. Significant differences compared with 0 h were identified by one-way ANOVA followed by Dunnett’s multiple comparisons test. **P < 0.01; ***P < 0.001. **G** IL-1β secretion levels in hBMECs pretreated with Gli or Z-VAD-FMK followed by 4 h of stimulation with rRfeA. The data are presented as the means ± s.d.; *n* = 3. Significant differences compared with no inhibitor were identified using one-way ANOVA followed by Dunnett’s multiple comparisons test. ***P < 0.001.

The release of lactate dehydrogenase (LDH) from cells is a well-established indicator of cellular damage [36]. Consequently, we utilized this method to evaluate the cytotoxic effects of rRfeA on hBMECs. Our results demonstrated that exposure to 10 µg/mL rRfeA for 4 h led to a significant increase in LDH release (Figure 2A). After 8 h of incubation, the LDH concentration in the cell culture supernatant surpassed 30 U/L (Figure 2A). Furthermore, the CCK8 assay, an additional technique for assessing cellular viability, supported these findings. Compared with that of the untreated control group (0 h), the viability of hBMECs treated with 10 µg/mL rRfeA decreased to approximately 70% after 8 h (Figure 2B). The RTX family of exoproteins is capable of forming pores in cell membranes, leading to cell death. However, spectrophotometric hemolytic assays demonstrated that rRfeA cannot damage the erythrocyte cytomembrane (Figure 2C). The NLRP3 inflammasome is a crucial component of the innate immune system, functioning as a sensor for a diverse array of exogenous and endogenous stimuli. Upon activation, the NLRP3 inflammasome promotes the activation of Caspase-1, a protease integral to the maturation and secretion of proinflammatory cytokines, such as interleukin-1β (IL-1β), as well as the induction of pyroptosis [37, 38]. To assess the ability of rRfeA to induce pyroptosis, hBMECs were treated with 10 µg/mL rRfeA, and samples were collected at 0–8 h post-treatment. First, western blot analysis was performed to quantify the protein levels of NLRP3 and Caspase-1-p20 in a time series experiment. Caspase-1 p20, a cleavage product of activated Caspase-1, serves as a biomarker for assessing Caspase-1 activation. The results demonstrated that the protein levels of NLRP3 and Caspase-1-p20 peaked at 2 h and then gradually decreased. For Caspase-1-p20, no significant difference was observed after 8 h (Figures 2D, E). To further confirm pyroptosis, the level of IL-1β secreted by hBMECs into the cell culture medium was measured. The concentration of IL-1β in the supernatant increased following rRfeA incubation, eventually surpassing 60 pg/mL (Figure 2F). Treatment with glibenclamide (Gli) and Z-YVAD-FMK, which specifically inhibit NLRP3 and Caspase-1, respectively, resulted in a reduction in IL-1β release (Figure 2G), indicating that rRfeA-induced pyroptosis occurs via the NLRP3/Caspase-1 pathway.

### RfeA-induced pyroptosis is dependent on mitochondrial reactive oxygen species

**Figure 3:**
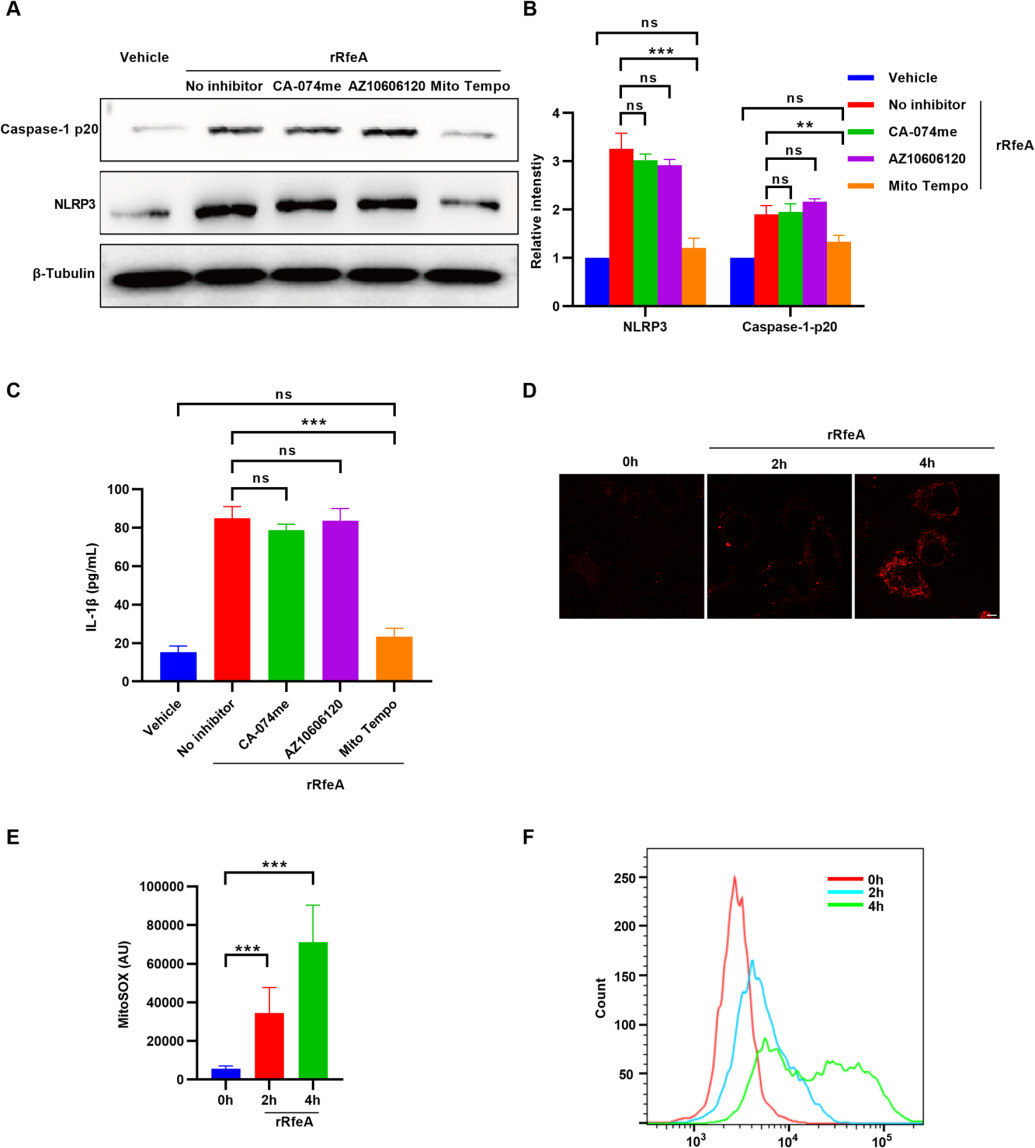
**RfeA activates the NLRP3/Caspase-1 pathway through mtROS**. **A** Representative western blots of the NLRP3 and Caspase-1 p20 proteins in hBMECs pretreated with CA-074me, AZ10606120, or MitoTempo followed by 4 h of rRfeA stimulation. β-Tubulin served as a loading control. **B** Quantitative analysis of NLRP3 and Caspase-1 p20 intensities in panel A. Vehicle-treated cells were used as a reference (1.0). The data are presented as the means ± s.d.; *n* = 3. Significant differences compared with no inhibitor were identified by two-way ANOVA followed by Dunnett’s multiple comparisons test. **P < 0.01; ***P < 0.001. **C** ELISA quantification of IL-1β secretion in culture supernatants from hBMECs pretreated with inhibitors (CA-074me, AZ10606120, MitoTempo) prior to 4 h of rRfeA stimulation. The data are presented as the means ± s.d.; *n* = 3. Significant differences compared with no inhibitor were identified by one-way ANOVA followed by Dunnett’s multiple comparisons test. ***P < 0.001. **D** Confocal microscopy images depicting mtROS accumulation in hBMECs at 0, 2, and 4 h post-rRfeA stimulation subjected to mitoSOX staining. Scale bar: 5 μm. **E** Quantitative analysis of the mean fluorescence intensity of mitoSOX. X-axis: different incubation times; Y-axis: MFI (arbitrary units). The data are presented as the means ± s.d.; *n* = 52 images. Significant differences compared with 0 h were identified by one-way ANOVA followed by Dunnett’s multiple comparisons test. ***P < 0.001. **F** Flow cytometric analysis of mtROS levels in hBMECs at 0, 2, and 4 h postrRfeA stimulation after mitoSOX staining.

To elucidate the pathway implicated in RfeA-induced pyroptosis, the inhibitors CA-074me, AZ10606120, and Mito Tempo were employed to suppress Cathepsin B activity, P2X7R activation, and mtROS accumulation, respectively. Notably, Mito Tempo treatment significantly attenuated the rRfeA-induced increase of NLRP3 and Caspase-1-p20 protein levels compared to the rRfeA-treated group without inhibitor (Figures 3A, B). In contrast, inhibition of Cathepsin B or P2X7R with CA-074me or AZ10606120, respectively, did not significantly alter the increased protein levels of NLRP3 and Caspase-1-p20 induced by rRfeA (Figures 3A, B). Furthermore, hBMECs pretreated with Mito Tempo exhibited decreased secretion of IL-1β compared to the rRfeA-treated group without inhibitor. However, CA-074me and AZ10606120 had no impact on the release of IL-1β in the cell culture medium following rRfeA stimulation (Figure 3C). To ascertain whether rRfeA induces mtROS production, mitoSOX was utilized to label intracellular mtROS following rRfeA treatment. As depicted in Figure 3D and E, rRfeA stimulation resulted in a fivefold increase in mtROS after 2 h, which escalated to over a tenfold increase after 4 h under confocal microscope. Flow cytometry analysis corroborated these findings, demonstrating an elevation in mtROS levels following rRfeA stimulation within a 4-h timeframe (Figure 3F).

### RfeA interacts with VDAC1 to stimulate the production of mtROS

**Figure 4:**
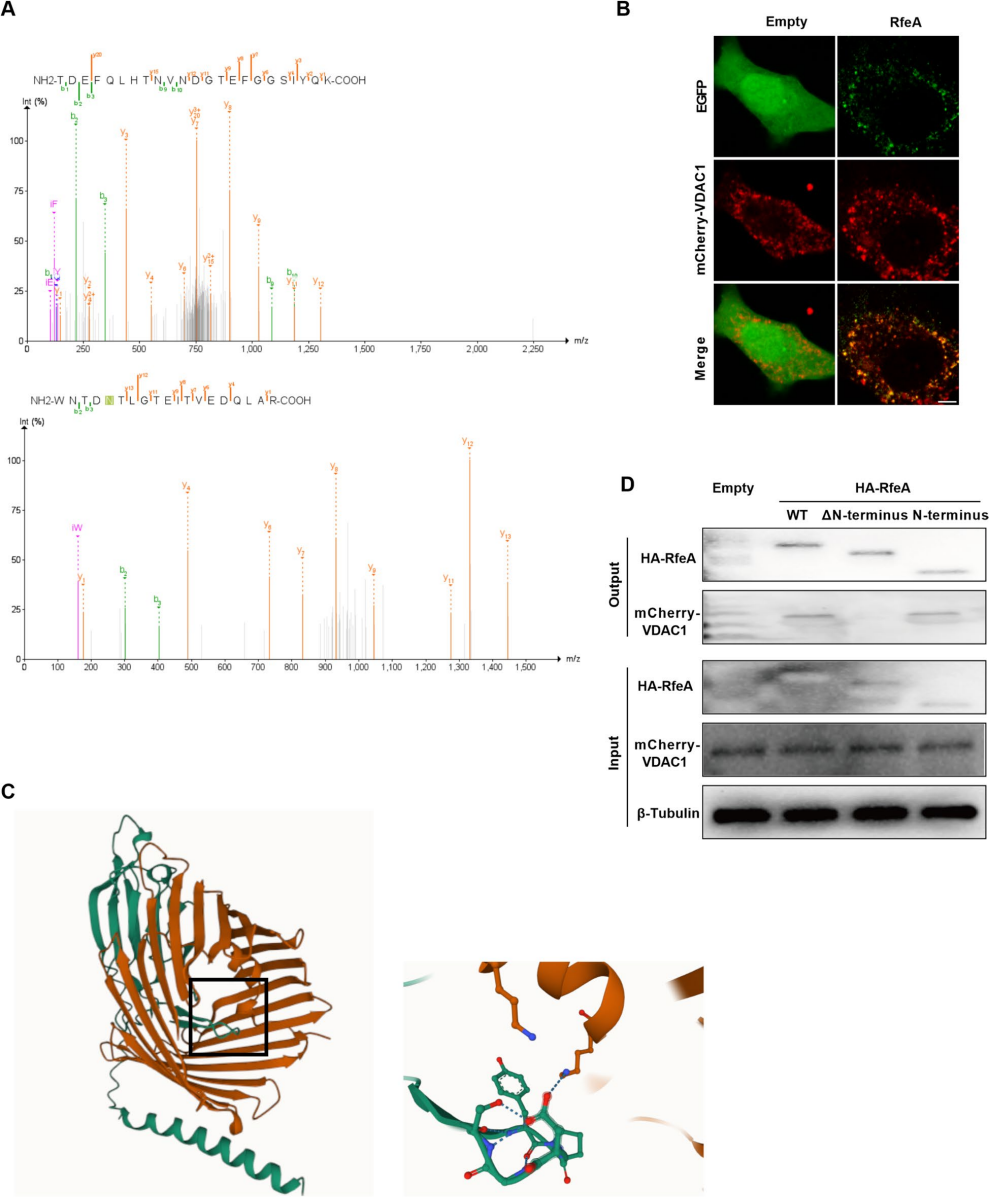
**RfeA N-terminus interacts with VDAC1. A** Mass spectrometry analysis identified two unique VDAC1 peptides (TDEFQLHTNVNDGTEFGGSIYQK and WNTDNTLGTEITVEDQLAR) in HA-RfeA immunoprecipitates from hBMEC lysates. **B** Representative confocal microscopy image showing the colocalization of VDAC1 (red) with EGFP-RfeA (green) in hBMECs but not EGFP alone. Scale bar: 5 μm. **C** AlphaFold3 forecasts the interaction between RfeA and VDAC1, with RfeA labelled in green and VDAC1 labelled in orange. **D** Representative western blots showing coimmunoprecipitation of VDAC1 with RfeA constructs: HA-RfeA-WT, HA-RfeA-ΔN-terminus and HA-RfeA-N-terminus. hBMECs transfected with empty vector (empty) were used as blank controls. β-Tubulin served as a loading control.

To elucidate the mechanism underlying mtROS production induced by RfeA, HA-RfeA was ectopically expressed in hBMECs and subsequently immunoprecipitated using beads conjugated with anti-HA antibodies. Following this, mass spectrometry analysis was conducted to identify proteins interacting with RfeA. In this study, we identified 63 proteins (Additional file 2), each characterized by the presence of at least two unique peptides, with a particular emphasis on proteins associated with mitochondria. Among these, the Voltage-Dependent Anion Channel 1 (VDAC1) is a pivotal protein situated in the outer mitochondrial membrane. Its strategic positioning enables interactions with various proteins, thereby influencing cellular survival and apoptotic pathways [39]. In our analysis, two unique peptides of VDAC1 (TDEFQLHTNVNDGTEFGGSIYQK and WNTDNTLGTEITVEDQLAR) were detected in concentrated HA-RfeA from hBMECs (Figure 4A). Confocal microscopy revealed a strong colocalization of mCherry-VDAC1 with EGFP-RfeA, whereas no colocalization was observed with EGFP alone (Figure 4B). Utilizing AlphaFold3 to predict the interaction between the two proteins, as depicted in Figure 4C, the interaction model reveals the presence of interaction sites between them. To further validate the interaction between VDAC1 and RfeA, Co-IP analysis was conducted. The results demonstrated detectable mCherry-VDAC1 immunoprecipitation signals in cells expressing HA-RfeA, while no such signals were present in cells lacking HA-RfeA expression, thereby confirming the interaction between RfeA and VDAC1 (Figure 4D). The predictive model indicates that the interaction between the N-terminal region of RfeA (1–27 aa) and VDAC1 is of critical importance, which was further elucidated. Our Co-IP analysis demonstrated that an RfeA mutant lacking the N-terminus (RfeA-ΔN-terminus) was unable to interact to mCherry-VDAC1 (Figure 4D). Notably, the ectopically expressed N-terminus of RfeA (RfeA-N-terminus), in isolation from other amino acid sequences, retained the ability to interact with mCherry-VDAC1 (Figure 4D). This observation indicates that the interaction between RfeA and VDAC1 is mediated specifically through the N-terminus of RfeA.

**Figure 5:**
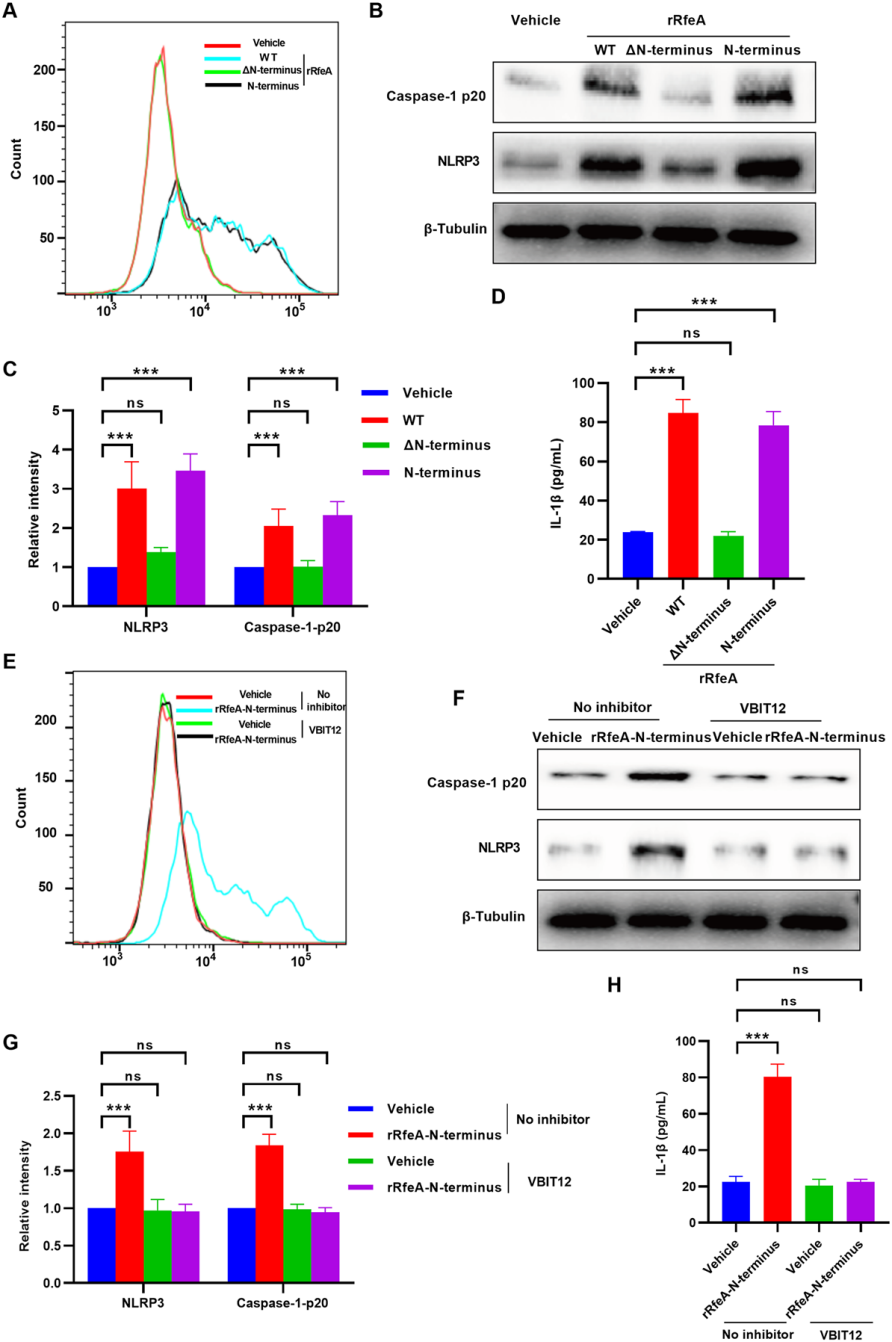
**The N-terminus of RfeA modulates mtROS production through interaction with VDAC1. A** Flow cytometric analysis of mtROS levels in hBMECs treated with vehicle (PBS) or rRfeA constructs: rRfeA-WT, rRfeA-ΔN-terminus and rRfeA-N-terminus followed by mitoSOX staining. **B** Representative western blot showing NLRP3 and Caspase-1 p20 bands in hBMECs treated with vehicle (PBS), rRfeA-WT, rRfeA-ΔN-terminus or rRfeA-N-terminus for 4 h. β-Tubulin served as a loading control. **C** Quantitative analysis of the gray values of the NLRP3 and Caspase-1 p20 protein bands in panel B. Vehicle (PBS)-treated cells were used as a reference (1.0). The data are presented as the means ± s.d.; *n* = 3. Significant differences compared with the vehicle group were identified via two-way ANOVA followed by Dunnett’s multiple comparisons test. ***P < 0.001. **D** ELISA-measured IL-1β secretion levels in culture supernatants from vehicle (PBS)- and rRfeA-treated hBMECs for 4 h. Three rRfeA constructs (rRfeA-WT, rRfeA-ΔN-terminus and rRfeA-N-terminus) were applied. The data are presented as the means ± s.d.; *n* = 3. Significant differences compared with the vehicle group were identified by one-way ANOVA followed by Dunnett’s multiple comparisons test. ***P < 0.001. **E** Flow cytometric analysis of mtROS in hBMECs pretreated with VBIT-12 followed by 4 h of rRfeA-N-terminus or vehicle (PBS) treatment by mitoSOX staining. **F** Representative western blots of NLRP3 and Caspase-1 p20 in hBMECs treated with VBIT-12 followed by the addition of rRfeA-N-terminus or vehicle (PBS) for 4 h. β-Tubulin served as a loading control. **G** Quantitative analysis of the intensities of the NLRP3 and Caspase-1 p20 bands in panel F. Vehicle (PBS) was set as 1.0. The data are presented as the means ± s.d.; *n* = 3. Significant differences compared with the vehicle group were identified by two-way ANOVA followed by Dunnett’s multiple comparisons test. ***P < 0.001. **H** ELISA-measured IL-1β secretion levels in hBMEC supernatants after VBIT-12 pretreatment and 4-h rRfeA-N-terminus or vehicle (PBS) stimulation. The data are presented as the means ± s.d.; *n* = 3. Significant differences compared with the vehicle group were identified by one-way ANOVA followed by Dunnett’s multiple comparisons test. ***P < 0.001.

To investigate the role of the RfeA-VDAC1 interaction in mtROS production, hBMECs were stimulated with purified rRfeA-WT, rRfeA-ΔN-terminus and rRfeA-N-terminus for 4 h, respectively. Flow cytometry analysis of mitoSOX revealed that the rRfeA-ΔN-terminus lost its ability to induce mtROS production, whereas the rRfeA N-terminus continued to promote mtROS generation, comparable to rRfeA-WT (Figure 5A). This suggests that the interaction involving RfeA-N-terminus is crucial for mtROS production. Subsequent western blot and ELISA analyses demonstrated that rRfeA-ΔN-terminus did not lead to the increase of protein levels of NLRP3, Caspase-1-p20, or the release of IL-1β (Figures 5B, C, D). In contrast, the rRfeA-N-terminus retained the capacity to induce these processes similarly to rRfeA-WT (Figures 5B, C, D). Given that VDAC1 oligomerization is associated with mtROS production [40], hBMECs were treated with VBIT-12, an inhibitor of VDAC1 oligomerization, prior to stimulation with rRfeA-N-terminus. mitoSOX staining showed that VBIT-12 significantly inhibited mtROS production after the addition of rRfeA-N-terminus (Figure 5E). Western blot results indicated that VBIT-12 also suppressed the increase in NLRP3 and Caspase-1-p20 levels induced by rRfeA-N-terminus (Figures 5F, G). Additionally, VBIT-12 inhibited the release of IL-1β following a 4-h incubation with rRfeA-N-terminus in hBMECs (Figure 5H). These findings suggest that the interaction between the N-terminus of RfeA and VDAC1 is integral to the observed biological effects.

### Pyroptosis induced by RfeA disrupts the blood–brain barrier

**Figure 6:**
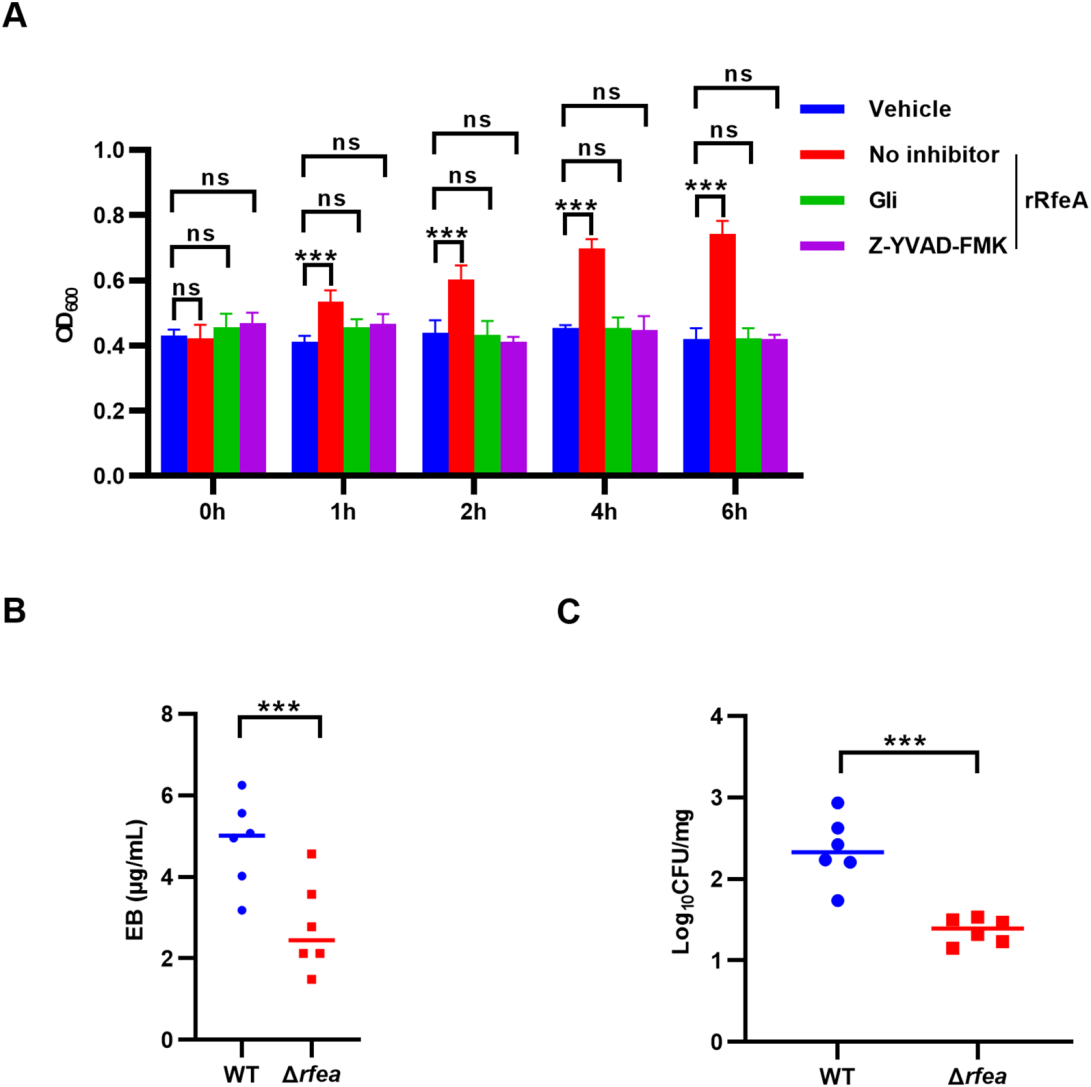
**RfeA-induced pyroptosis disrupts blood‒brain barrier integrity**. **A** Assessment of the integrity of hBMEC monolayers pretreated with Gli or Z-YVAD-FMK followed by the addition of rRfeA or vehicle (PBS) via an Evans blue penetration assay. The data are presented as the means ± s.d.; *n* = 3. Significant differences compared with the vehicle group were identified by two-way ANOVA followed by Tukey’s multiple comparisons test. ***P < 0.001. **B** Evans blue dye penetration assay in the brains of C57BL/6 J mice challenged with the WT strain or the Δ*rfea* strain. The horizontal lines represent the geometric means. Significant differences were identified via paired t tests. ***P < 0.001. **C** Bacterial load in the brains of mice 2 days following infection with the WT or Δ*rfea* SS2 strains. The horizontal lines represent the geometric means. Significant differences were identified via paired t tests. ***P < 0.001.

Bacterial components are able to induce pyroptosis to disrupt BBB [41], thus, we hypothesize that RfeA-induced pyroptosis attributes to disruption of BBB. Evans blue permeability assay was conducted to identify the impact of RfeA on the integrity of hBMEC monolayers. Addition of rRfeA led to a time-dependent increase in barrier permeability, which was not observable after treatment of Gli or Z-YVAD-FMK (Figure 6A). To further analyse the impact of RfeA on BBB, *rfea* deletion mutant (Δ*rfea*) was generated. In addition, then C57 mice were challenged with the WT strain and the Δ*rfea* strain to evaluate the permeability of BBB in vivo*.* The brains of mice infected with WT SS2 exhibited significantly higher levels of detectable EB compared to those infected with the Δ*rfea* strain (Figure 6B). To identify whether RfeA affects the ability of SS2 to enter brain, bacterial loads in brains were assessed 2 days following infection. Bacterial burdens in brains for the SS2 Δ*rfea* strain were significantly lower than those for WT strain (Figure 6C).

## Discussion

The blood–brain barrier functions as a vital protective mechanism, safeguarding the central nervous system from potentially harmful pathogens, including SS2. However, the precise mechanisms by which SS2 penetrates this barrier remain unclear. In this study, we demonstrated that the RTX family exoprotein A, which is secreted by SS2, can induce pyroptosis in hBMECs, leading to disruption of the BBB. Further analysis revealed that RfeA-induced pyroptosis is dependent on mtROS. RfeA has the ability to interact with VDAC1, resulting in the production of mtROS, which can be impeded by the inhibition of VDAC1 oligomerization. The N-terminus of RfeA is essential for the interaction between RfeA and VDAC1. This discovery regarding host‒bacteria interactions enhances our understanding of BBB breakdown and identifies a novel SS2 virulence factor that contributes to meningitis.

The RTX (repeats-in-toxin) family of exoproteins constitutes a diverse group of proteins secreted by bacteria, distinguished by their capacity to form pores in host cell membranes, thereby inducing cytolytic and cytotoxic effects [42]. These proteins serve as significant virulence factors that increase the pathogenicity of various bacterial species, which are predominantly identified in gram-negative bacteria [43]. A well-studied member of this family is adenylate cyclase toxin (CyaA) from *Bordetella pertussis*, which plays a pivotal role in the pathogenesis of whooping cough. CyaA is recognized for its ability to invade host cells and disrupt cellular signalling by converting ATP to cyclic AMP (cAMP), thereby impairing immune responses [44]. Additionally, RfeA has been identified in SS2, and purified RfeA has been shown to induce effective protection against SS2 infection in mice [33]; however, the role of RfeA in SS2 infection remains to be elucidated. In this study, we identified the capacity of RfeA to induce pyroptosis in hBMECs, subsequently leading to disruption of the BBB. Our investigation focused exclusively on the toxic effects of RfeA on hBMECs, and further research is necessary to determine whether RfeA affects other cell types. Upon binding to eukaryotic cells, CyaA is internalized through its interaction with calmodulin [35]. Our findings indicate that the use of a calmodulin inhibitor does not impede the entry of RfeA into hBMECs; rather, RfeA is internalized via a caveolae/lipid raft-dependent endocytic pathway. RTX toxins, such as CyaA, are secreted through the type I secretion system (T1SS), an efficient mechanism that facilitates the translocation of proteins across the bacterial cell envelope in a single step [45]. However, SS2 lacks this secretion system, necessitating further investigation into the secretory pathway of RfeA. Typically, RTX toxins insert their hydrophobic segments into the lipid bilayer to form transmembrane pores, disrupting ionic gradients and membrane potential, ultimately leading to cell death [46]. In contrast, RfeA does not exhibit cytolytic activity toward red blood cells, indicating that RfeA secreted by SS2 lacks some canonical ability of RTX toxins.

Pyroptosis, an inflammatory variant of programmed cell death, is integral to the host defense mechanism against bacterial infections. This process not only eradicates the replicative environment of intracellular pathogens but also facilitates the release of proinflammatory cytokines, including IL-1β and IL-18, thereby enhancing the immune response [47]. Recent studies have highlighted the role of pyroptosis in compromising the integrity of the BBB. Bacterial lipopolysaccharide (LPS) has been shown to activate inflammatory caspases and gasdermin D (GSDMD) in brain endothelial cells, leading to pyroptotic cell death and subsequent disruption of the BBB in a mouse model [48]. This process underscores the potential of pyroptosis for bacteria to penetrate the BBB, leading to severe CNS infections such as meningitis. Pyroptosis is involved in SS2 peptidyl-prolyl isomerase (PrsA)-induced cell death [49], and our study identified a new component of SS2 that induces proptosis in brain endothelial cells, with the increase in mtROS mediated by RfeA being critical for this process. mtROS are crucial in regulating pyroptosis. Elevated mtROS levels can activate the NLRP3 inflammasome, leading to the conversion of pro-caspase-1 into its active form. This activation subsequently results in the cleavage of GSDMD, which forms pores in the cell membrane, causing cell swelling, lysis, and the release of proinflammatory cytokines such as IL-1β [38]. Polyphenol-mediated antioxidase complexes efficiently scavenge excess mtROS and reverse mitochondrial depolarization, thereby inhibiting NLRP3-mediated pyroptosis [50]. This approach highlights the potential of targeting mitochondrial oxidative stress as a therapeutic strategy to mitigate pyroptosis and alleviate the BBB disruption induced by RfeA.

VDAC1 is a crucial protein located in the outer mitochondrial membrane that plays a significant role in regulating mitochondrial function and cellular metabolism [51]. VDAC1 is known to interact with various proteins and pathways that influence mtROS levels. For example, the interaction between VDAC1 and mitoNEET, a redox-sensitive protein, has been shown to regulate the gating of VDAC1 in a redox-dependent manner [52]. This interaction affects the flow of metabolites and ions, thereby influencing mtROS production and mitochondrial function [52]. Our research demonstrated that the virulent protein of bacteria can directly interact with VDAC1, resulting in increased intracellular mtROS levels. The application of a VDAC1 oligomerization inhibitor effectively suppressed the increase in mtROS and the subsequent induction of pyroptosis.

In conclusion, this study elucidates a new function of RfeA in bacterial pathogenesis. The identification of the interaction between RfeA and the mitochondrial protein VDAC1 as a mechanism for inducing pyroptosis and increasing BBB permeability significantly broadens our understanding of the strategies employed by bacterial virulence factors to cause meningitis.

## Supplementary Information

**Additional file 1. Details of the primers used in this study.**

**Additional file 2. List of proteins identified to interact with RfeA via LC‒MS/MS analysis.**

## Data Availability

The datasets analysed during the current study are available from the corresponding author upon reasonable request.

## References

[1] Feng Y, Zhang H, Ma Y, Gao GF (2010) Uncovering newly emerging variants of *Streptococcus suis*, an important zoonotic agent. Trends Microbiol 18:124–131 20071175 10.1016/j.tim.2009.12.003

[2] Segura M, Fittipaldi N, Calzas C, Gottschalk M (2017) Critical *Streptococcus suis* virulence factors: are they all really critical? Trends Microbiol 25:585–599 28274524 10.1016/j.tim.2017.02.005

[3] Segura M (2020) *Streptococcus suis* research: progress and challenges. Pathogens 9:707 32867188 10.3390/pathogens9090707 PMC7557840

[4] Thanh NH, Huy DT, Anh VTT, Anh NN, Uyen NTT, Hanh NHH, Phu NH (2024) *Streptococcus suis*-associated meningitis in a southern region of Vietnam. Am J Trop Med Hyg 111:1247–1251 39406252 10.4269/ajtmh.23-0774 PMC11619494

[5] Kerdsin A (2022) Human *Streptococcus suis* infections in Thailand: epidemiology, clinical features, genotypes, and susceptibility. Trop Med Infect Dis 7:359 36355901 10.3390/tropicalmed7110359 PMC9695567

[6] Susilawathi NM, Tarini NMA, Fatmawati NND, Mayura PIB, Suryapraba AAA, Subrata M, Sudewi AAR, Mahardika GN (2019) *Streptococcus suis*-associated meningitis, Bali, Indonesia, 2014–2017. Emerg Infect Dis 25:2235–2242 31742523 10.3201/eid2512.181709 PMC6874276

[7] Wertheim HF, Nghia HD, Taylor W, Schultsz C (2009) *Streptococcus suis*: an emerging human pathogen. Clin Infect Dis 48:617–625 19191650 10.1086/596763

[8] Guntala R, Khamai L, Srisai N, Ounjaijean S, Khamduang W, Hongjaisee S (2024) Contamination of *Streptococcus suis* and *S. suis* serotype 2 in raw pork and edible pig organs: a public health concern in Chiang Mai, Thailand. Foods 13:2119 38998625 10.3390/foods13132119 PMC11241745

[9] Ji L, Chen Z, Li F, Hu Q, Xu L, Duan X, Wu H, Xu S, Chen Q, Wu S, Qiu S, Lu H, Jiang M, Cai R, Qiu Y, Li Y, Shi X (2023) Epidemiological and genomic analyses of human isolates of *Streptococcus suis* between 2005 and 2021 in Shenzhen, China. Front Microbiol 14:1118056 37113229 10.3389/fmicb.2023.1118056 PMC10126776

[10] Ibrahim A, Saleem N, Naseer F, Ahmed S, Munawar N, Nawaz R (2024) From cytokines to chemokines: understanding inflammatory signaling in bacterial meningitis. Mol Immunol 173:117–126 39116800 10.1016/j.molimm.2024.07.004

[11] Takeuchi D, Akeda Y, Nakayama T, Kerdsin A, Sano Y, Kanda T, Hamada S, Dejsirilert S, Oishi K (2014) The contribution of suilysin to the pathogenesis of *Streptococcus suis* meningitis. J Infect Dis 209:1509–1519 24285845 10.1093/infdis/jit661

[12] Jiang H, Sun Y, Li F, Yu X, Lei S, Du S, Wu T, Jiang X, Zhu J, Wang J, Ji Y, Li N, Feng X, Gu J, Han W, Zeng L, Lei L (2024) Enolase of *Streptococcus suis* serotype 2 promotes biomolecular condensation of ribosomal protein SA for HBMECs apoptosis. BMC Biol 22:33 38331785 10.1186/s12915-024-01835-y PMC10854124

[13] Liu H, Lei S, Jia L, Xia X, Sun Y, Jiang H, Zhu R, Li S, Qu G, Gu J, Sun C, Feng X, Han W, Langford PR, Lei L (2021) *Streptococcus suis* serotype 2 enolase interaction with host brain microvascular endothelial cells and RPSA-induced apoptosis lead to loss of BBB integrity. Vet Res 52:30 33618766 10.1186/s13567-020-00887-6 PMC7898445

[14] Kong D, Chen Z, Wang J, Lv Q, Jiang H, Zheng Y, Xu M, Zhou X, Hao H, Jiang Y (2017) Interaction of factor H-binding protein of *Streptococcus suis* with globotriaosylceramide promotes the development of meningitis. Virulence 8:1290–1302 28402705 10.1080/21505594.2017.1317426 PMC5711355

[15] Pan Z, He P, Zhang Y, Gu Q, Chen S, Yu Y, Shao J, Wang K, Wu Z, Yao H, Ma J (2022) SssP1, a fimbria-like component of *Streptococcus suis*, binds to the vimentin of host cells and contributes to bacterial meningitis. PLoS Pathog 18:e1010710 35853077 10.1371/journal.ppat.1010710 PMC9337661

[16] Xu L, Lu X, Xiao P, Liu R, Xia K, Wu M, Jin M, Zhang A (2022) Interleukin-17A contributed to the damage of blood-CNS barriers during *Streptococcus suis* meningitis. Mol Neurobiol 59:2116–2128 35044625 10.1007/s12035-022-02749-y

[17] Jorgensen I, Miao EA (2015) Pyroptotic cell death defends against intracellular pathogens. Immunol Rev 265:130–142 25879289 10.1111/imr.12287 PMC4400865

[18] Junqueira C, Crespo Â, Ranjbar S, de Lacerda LB, Lewandrowski M, Ingber J, Parry B, Ravid S, Clark S, Schrimpf MR, Ho F, Beakes C, Margolin J, Russell N, Kays K, Boucau J, Das Adhikari U, Vora SM, Leger V, Gehrke L, Henderson LA, Janssen E, Kwon D, Sander C, Abraham J, Goldberg MB, Wu H, Mehta G, Bell S, Goldfeld AE, Filbin MR, Lieberman J (2022) Fcγr-mediated SARS-CoV-2 infection of monocytes activates inflammation. Nature 606:576–584 35385861 10.1038/s41586-022-04702-4 PMC10071495

[19] Ding J, Shao F (2017) SnapShot: the noncanonical inflammasome. Cell 168:544-544.e1 28129545 10.1016/j.cell.2017.01.008

[20] Braga TT, Forni MF, Correa-Costa M, Ramos RN, Barbuto JA, Branco P, Castoldi A, Hiyane MI, Davanso MR, Latz E, Franklin BS, Kowaltowski AJ, Camara NO (2017) Soluble uric acid activates the nLRP3 inflammasome. Sci Rep 7:39884 28084303 10.1038/srep39884 PMC5233987

[21] Wang L, Chen Y, Li X, Zhang Y, Gulbins E, Zhang Y (2016) Enhancement of endothelial permeability by free fatty acid through lysosomal cathepsin B-mediated Nlrp3 inflammasome activation. Oncotarget 7:73229–73241 27689324 10.18632/oncotarget.12302 PMC5341975

[22] Peng K, Liu L, Wei D, Lv Y, Wang G, Xiong W, Wang X, Altaf A, Wang L, He D, Wang H, Qu P (2015) P2X7R is involved in the progression of atherosclerosis by promoting NLRP3 inflammasome activation. Int J Mol Med 35:1179–1188 25761252 10.3892/ijmm.2015.2129 PMC4380202

[23] Paik S, Kim JK, Silwal P, Sasakawa C, Jo EK (2021) An update on the regulatory mechanisms of NLRP3 inflammasome activation. Cell Mol Immunol 18:1141–1160 33850310 10.1038/s41423-021-00670-3 PMC8093260

[24] Hughes CS, Colhoun LM, Bains BK, Kilgour JD, Burden RE, Burrows JF, Lavelle EC, Gilmore BF, Scott CJ (2016) Extracellular cathepsin S and intracellular caspase 1 activation are surrogate biomarkers of particulate-induced lysosomal disruption in macrophages. Part Fibre Toxicol 13:19 27108091 10.1186/s12989-016-0129-5 PMC4842290

[25] Yang D, He Y, Muñoz-Planillo R, Liu Q, Núñez G (2015) Caspase-11 requires the Pannexin-1 channel and the purinergic P2X7 pore to mediate pyroptosis and endotoxic shock. Immunity 43:923–932 26572062 10.1016/j.immuni.2015.10.009 PMC4795157

[26] Chandel NS (2021) Mitochondria. Cold Spring Harb Perspect Biol 13:a040543 33649187 10.1101/cshperspect.a040543 PMC7919390

[27] Shen X, Ran J, Yang Q, Li B, Lu Y, Zheng J, Xu L, Jia K, Li Z, Peng L, Fang R (2024) RACK1 and NEK7 mediate GSDMD-dependent macrophage pyroptosis upon *Streptococcus suis* infection. Vet Res 55:120 39334337 10.1186/s13567-024-01376-w PMC11428613

[28] Wang S, Wang G, Tang YD, Li S, Qin L, Wang M, Yang YB, Gottschalk M, Cai X (2022) *Streptococcus suis* serotype 2 infection induces splenomegaly with splenocyte apoptosis. Microbiol Spectr 10:e0321022 36287014 10.1128/spectrum.03210-22 PMC9769541

[29] Xu L, Lin L, Lu X, Xiao P, Liu R, Wu M, Jin M, Zhang A (2021) Acquiring high expression of suilysin enable non-epidemic *Streptococccus suis* to cause streptococcal toxic shock-like syndrome (STSLS) through NLRP3 inflammasome hyperactivation. Emerg Microbes Infect 10:1309–1319 33792531 10.1080/22221751.2021.1908098 PMC8253218

[30] Lin L, Xu L, Lv W, Han L, Xiang Y, Fu L, Jin M, Zhou R, Chen H, Zhang A (2019) An NLRP3 inflammasome-triggered cytokine storm contributes to Streptococcal toxic shock-like syndrome (STSLS). PLoS Pathog 15:e1007795 31170267 10.1371/journal.ppat.1007795 PMC6553798

[31] Cao X, Jia K, Liu Q, Yin H, Yu X, Hu X, Ye C, Peng L, Fang R (2024) The critical role of NLRP3 inflammasome activation in *Streptococcus suis*-induced blood-brain barrier disruption. Vet Microbiol 295:110161 38945021 10.1016/j.vetmic.2024.110161

[32] Shi K, Li Y, Xu M, Zhang K, Gou H, Li C, Zhai S (2024) Membrane vesicles derived from *Streptococcus suis* serotype 2 induce cell pyroptosis in endothelial cells via the NLRP3/Caspase-1/GSDMD pathway. J Integr Agricult 23:1338–1353

[33] Liu L, Cheng G, Wang C, Pan X, Cong Y, Pan Q, Wang J, Zheng F, Hu F, Tang J (2009) Identification and experimental verification of protective antigens against *Streptococcus suis* serotype 2 based on genome sequence analysis. Curr Microbiol 58:11–17 18839251 10.1007/s00284-008-9258-x

[34] Che R, Ding S, Zhang Q, Yang W, Yan J, Lin X (2019) Haemolysin Sph2 of *Leptospira interrogans* induces cell apoptosis via intracellular reactive oxygen species elevation and mitochondrial membrane injury. Cell Microbiol 21:e12959 30278102 10.1111/cmi.12959

[35] Voegele A, Sadi M, O’Brien DP, Gehan P, Raoux-Barbot D, Davi M, Hoos S, Brûlé S, Raynal B, Weber P, Mechaly A, Haouz A, Rodriguez N, Vachette P, Durand D, Brier S, Ladant D, Chenal A (2021) A high-affinity calmodulin-binding site in the CyaA toxin translocation domain is essential for invasion of eukaryotic cells. Adv Sci (Weinh) 8:2003630 33977052 10.1002/advs.202003630 PMC8097335

[36] Kumar P, Nagarajan A, Uchil PD (2018) Analysis of cell viability by the lactate dehydrogenase assay. Cold Spring Harb Protoc 2018:e095497 10.1101/pdb.prot095497 29858337

[37] Fu J, Wu H (2023) Structural mechanisms of NLRP3 inflammasome assembly and activation. Annu Rev Immunol 41:301–316 36750315 10.1146/annurev-immunol-081022-021207 PMC10159982

[38] Huang Y, Xu W, Zhou R (2021) Nlrp3 inflammasome activation and cell death. Cell Mol Immunol 18:2114–2127 34321623 10.1038/s41423-021-00740-6 PMC8429580

[39] Shoshan-Barmatz V, Maldonado EN, Krelin Y (2017) VDAC1 at the crossroads of cell metabolism, apoptosis and cell stress. Cell Stress 1:11–36 30542671 10.15698/cst2017.10.104 PMC6287957

[40] Zhu X, Luo L, Xiong Y, Jiang N, Wang Y, Lv Y, Xie Y (2022) VDAC1 oligomerization may enhance DDP-induced hepatocyte apoptosis by exacerbating oxidative stress and mitochondrial DNA damage. FEBS Open Bio 12:516–522 34967508 10.1002/2211-5463.13359 PMC8804618

[41] VanHook AM (2024) Bacteria-induced BBB breakdown. Sci Signal 17:eadq7330 38833529 10.1126/scisignal.adq7330

[42] Ostolaza H, González-Bullón D, Uribe KB, Martín C, Amuategi J, Fernandez-Martínez X (2019) Membrane permeabilization by pore-forming RTX toxins: what kind of lesions do these toxins form? Toxins (Basel) 11:354 31216745 10.3390/toxins11060354 PMC6628442

[43] Wang H, Gao X, Li H (2019) Single molecule force spectroscopy reveals the mechanical design governing the efficient translocation of the bacterial toxin protein RTX. J Am Chem Soc 141:20498–20506 31786929 10.1021/jacs.9b11281

[44] Ahmad JN, Sebo P (2021) Bacterial RTX toxins and host immunity. Curr Opin Infect Dis 34:187–196 33899753 10.1097/QCO.0000000000000726

[45] Sotomayor-Pérez AC, Ladant D, Chenal A (2014) Disorder-to-order transition in the CyaA toxin RTX domain: implications for toxin secretion. Toxins (Basel) 7:1–20 25559101 10.3390/toxins7010001 PMC4303809

[46] González Bullón D, Uribe KB, Amuategi J, Martín C, Ostolaza H (2021) Cholesterol stimulates the lytic activity of adenylate cyclase toxin on lipid membranes by promoting toxin oligomerization and formation of pores with a greater effective size. FEBS J 288:6795–6814 34216517 10.1111/febs.16107 PMC9290974

[47] Maltez VI, Tubbs AL, Cook KD, Aachoui Y, Falcone EL, Holland SM, Whitmire JK, Miao EA (2015) Inflammasomes coordinate pyroptosis and natural killer cell cytotoxicity to clear infection by a ubiquitous environmental bacterium. Immunity 43:987–997 26572063 10.1016/j.immuni.2015.10.010 PMC4654968

[48] Wei C, Jiang W, Wang R, Zhong H, He H, Gao X, Zhong S, Yu F, Guo Q, Zhang L, Schiffelers LDJ, Zhou B, Trepel M, Schmidt FI, Luo M, Shao F (2024) Brain endothelial GSDMD activation mediates inflammatory BBB breakdown. Nature 629:893–900 38632402 10.1038/s41586-024-07314-2

[49] Jiang X, Yu G, Zhu L, Siddique A, Zhan D, Zhou L, Yue M (2023) Flanking N- and C-terminal domains of PrsA in *Streptococcus suis* type 2 are crucial for inducing cell death independent of TLR2 recognition. Virulence 14:2249779 37641974 10.1080/21505594.2023.2249779 PMC10467536

[50] Zhang J, Gao B, Ye B, Sun Z, Qian Z, Yu L, Bi Y, Ma L, Ding Y, Du Y, Wang W, Mao Z (2023) Mitochondrial-targeted delivery of polyphenol-mediated antioxidases complexes against pyroptosis and inflammatory diseases. Adv Mater 35:e2208571 36648306 10.1002/adma.202208571

[51] Shoshan-Barmatz V, Nahon-Crystal E, Shteinfer-Kuzmine A, Gupta R (2018) VDAC1, mitochondrial dysfunction, and Alzheimer’s disease. Pharmacol Res 131:87–101 29551631 10.1016/j.phrs.2018.03.010

[52] Lipper CH, Stofleth JT, Bai F, Sohn YS, Roy S, Mittler R, Nechushtai R, Onuchic JN, Jennings PA (2019) Redox-dependent gating of VDAC by mitoNEET. Proc Natl Acad Sci U S A 116:19924–19929 31527235 10.1073/pnas.1908271116 PMC6778226

