# Supplementary material for: RfeA from Streptococcus suis serotype 2 triggers NLRP3/Caspase-1-dependent pyroptosis leading to blood–brain barrier disruption
Source: Vet Res. 2025 Sep 25;56:184. doi: 10.1186/s13567-025-01620-x (PMC12465831; doi:10.1186/s13567-025-01620-x)
Supplement: Supplementary file 1 — Additional file 1. Details of the primers used in this study. [file 13567_2025_1620_MOESM1_ESM.docx]

**Additional file 1 Details of primers applied in this study.**

| Primer name | Sequence |
| --- | --- |
| Δrefa-A | cgcaagcttggcctcagaaatcaatgatgctaagact |
| Δrefa-B | gaggaagagcttatctgaagcatctttaga |
| Δrefa-C | tctaaagatgcttcagataagctcttcctc |
| Δrefa-D | cgcgaattcttcctaccgctttccttcatgattatgtac |
| 28a-F | cgccatatggtgacaattaaatatccagatggaactattgatttg |
| 28a-R | cgcggatccttaagcttcttcgtctcttcttctctttgc |
| EGFP-F | cgcagatctgtgacaattaaatatccagatggaactattgatttg |
| EGFP-R | cgcgaattcttaagcttcttcgtctcttcttctctttgc |
| 3HA-F | cgcggtaccgtgacaattaaatatccagatggaactattgatttg |
| 3HA-R | cgcggatccttaagcttcttcgtctcttcttctctttgc |
| 3HA-N-terminus-F | cgcggtaccgtgacaattaaatatccagatggaactattgat |
| 3HA-N-terminus-R | cgcggatccaggagcagttttatctgcctg |
| 3HA-ΔN-terminus-F | cgcggtaccacggtcgcaaatgatggcaa |
| 3HA-ΔN-terminus-R | cgcggatccttaagcttcttcgtctcttcttctctttgc |
| 28a-N-terminus-F | cgccatatggtgacaattaaatatccagatggaactattgat |
| 28a-N-terminus-R | cgcggatccaggagcagttttatctgcctg |
| 28a-ΔN-terminus-F | cgccatatgacggtcgcaaatgatggcaa |
| 28a-ΔN-terminus-R | cgcggatccttaagcttcttcgtctcttcttctctttgc |
